# Language Lateralization by Passive Auditory fMRI in Presurgical Assessment for Temporal Lobe Epilepsy: A Single-Center Retrospective Study

**DOI:** 10.3390/jcm13061706

**Published:** 2024-03-15

**Authors:** Yoji Okahara, Kyoko Aoyagi, Hiroto Iwasa, Yoshinori Higuchi

**Affiliations:** 1 Department of Neurological Surgery, Chiba Cerebral and Cardiovascular Center, Chiba 290-0512, Japanyhiguchi@faculty.chiba-u.jp (Y.H.); 2 Kisarazu Epilepsy Center, Kisarazu Hospital, Chiba 292-8535, Japan; 3 Department of Neurological Surgery, Chiba University, Chiba 263-8522, Japan

**Keywords:** epilepsy, Wada test, functional MRI, language lateralization, passive auditory stimuli

## Abstract

**Background**: In temporal lobe epilepsy (TLE), estimating the potential risk of language dysfunction before surgery is a necessary procedure. Functional MRI (fMRI) is considered the most useful to determine language lateralization noninvasively. However, there are no standardized language fMRI protocols, and several issues remain unresolved. In particular, the language tasks normally used are predominantly active paradigms that require the overt participation of patients, making assessment difficult for pediatric patients or patients with intellectual disabilities. In this study, task-based fMRI with passive narrative listening was applied to evaluate speech comprehension to estimate language function in Japanese-speaking patients with drug-resistant TLE. **Methods**: Twenty-one patients (six with intellectual disabilities) participated. Patients listened to passive auditory stimuli with combinations of forward and silent playback, and forward and backward playback. The activation results were extracted using a block design, and lateralization indices were calculated. The obtained fMRI results were compared to the results of the Wada test. **Results:** The concordance rate between fMRI and the Wada test was 95.2%. Meaningful responses were successfully obtained even from participants with intellectual disabilities. **Conclusions:** This passive fMRI paradigm can provide safe and easy presurgical language evaluation, particularly for individuals who may not readily engage in active paradigms.

## 1. Introduction

Frequent epileptic seizures disrupt language networks and promote brain plasticity [1,2]. Several reports have suggested that atypical language distributions, such as bilateral or right-dominant language lateralization, are more frequently observed in patients with temporal lobe epilepsy (TLE) than in healthy controls [3,4,5]. The incidence of language dysfunction after anterior temporal lobectomy (ATL) is known to be different in the language-dominant hemisphere than that in the language-non-dominant hemisphere; left ATL causes some degree of worsening in naming and reading in 34–40% of patients, while right ATL causes a decline in these functions in only 5% of the cases [6,7]. As language deficits can significantly affect the quality of life [8], detecting the language processing area preceding the epileptic surgery is a critical procedure.

Several approaches have been used to clinically identify specific cortices, including electrical stimulation mapping (ESM), transcranial magnetic stimulation [9], functional magnetic resonance imaging (fMRI) [10], and resting-state functional MRI (rfMRI) [11]. Mapping for language localization using ESM is the most accurate method; however, it requires a craniotomy and active patient participation [12,13]. Although the value of preoperative fMRI mapping is fairly consistent [14,15,16], fMRI tends to show all involved language regions, which are not necessarily essential for language function [17], and it is not the first choice for language localization.

There are two representative methods for estimating language lateralization: the intracarotid amobarbital test (Wada test) [18] and fMRI. The Wada test was once the gold standard method for determining language lateralization but has now been replaced by fMRI, because of its invasiveness and limitations, in the majority of comprehensive epilepsy centers [19]. fMRI has great potential not only to determine the dominant hemispheres associated with language but also to delineate language-associated brain areas. There are two main types of preoperative assessments that use fMRI: language production and language comprehension tasks. Language production tasks include object naming (visual or auditory) and verb generation, whereas representative language comprehension tasks are sentence comprehension or rhyme detection. As most of these tasks are active paradigms that require participants to respond to speech sounds and understand verbal instructions, they are less suitable for younger children and developmentally disabled participants. This creates an urgent demand for reliable functional language mapping methods for characteristically non-compliant patients.

Based on the aforementioned context, the necessity for functional language assessment through a passive paradigm became evident, leading to the establishment of our research focus. Our research question emerged from the recognition of a critical need in assessing language lateralization and speech comprehension in Japanese-speaking patients with drug-resistant epilepsy requiring epileptic surgery. The focus shifted towards evaluating the efficacy of passive narrative-listening tasks utilizing fMRI as an alternative method. To detect passive neural responses, we developed passive narrative-listening tasks using fMRI. These tasks consisted of two speech conditions that allowed the evaluation of speech comprehension. Our previous report confirmed that the developed task could elicit meaningful responses in patients with unresponsive wakefulness syndrome [20]. The purpose of this study was to demonstrate a passive auditory fMRI paradigm capable of accurately assessing speech comprehension and establishing correlations with the Wada test outcomes in Japanese-speaking patients with epilepsy. This study may lead to the proposal of a non-invasive alternative method for evaluating language lateralization for presurgical assessments in patients with TLE.

## 2. Materials and Methods

### 2.1. Participants

Of the 716 patients referred to Chiba Cerebral and Cardiovascular Center (which is the only institution of comprehensive epilepsy center in Chiba prefecture) between August 2018 and June 2023, 23 patients with temporal lobe epilepsy (TLE) who met inclusion criteria were recruited retrospectively. Inclusion criteria in this study were as follows: a participant who was diagnosed with drug-resistant temporal lobe epilepsy with no structural abnormalities within areas of the temporal and inferior parietal cortices, had no history of brain surgery, gave consent to proceed to future surgical treatment for epilepsy, and was a native Japanese speaker. We defined drug-resistant epilepsy as failure of adequate trials of two tolerated and appropriately chosen and used anti-epileptic drug schedules, following the guideline by the International League Against Epilepsy [21]. A 3 Tesla MRI was performed to detect brain lesions, which were checked by two independent board-certified neurosurgeons. We excluded two participants who could not tolerate the MRI scan after two attempts; one had a lot of head movements and the other was scared to hear the auditory stimuli in this experiment. Thus, 21 patients were included in this study (8 men and 13 women; mean age, 38.6 years old; 18 right-handed). All patients underwent neuropsychological examinations, including the Mini-Mental State Examination (MMSE) and the Wechsler Adult Intelligence Scale 3rd edition (WAIS-III). All neuropsychological testing was performed by a Japanese-certified speech–language–hearing therapist. The clinical diagnosis of epilepsy was evaluated based on noninvasive data, including clinical history, risk factors for epilepsy, possible etiology, seizure semiology, interictal and ictal video scalp-EEG findings by the results of long-term video video-electroencephalography monitoring (VTREEG), MRI, and neuropsychological assessment. We estimated the side of the epileptic focus by the following assessments: VTREEG, fluorodeoxyglucose-positron emission tomography, and electroencephalography-correlated fMRI. All processes were finally confirmed by a board-certified epileptologist from the Japan Epilepsy Society, followed by an interactive discussion in a roundtable conference to reach a general consensus. The clinical features of the patients are summarized in Table 1.

Informed consent was obtained from all patients, or their families, following the institutional guidelines, and the institutional ethics committee approved the present study protocol (IRB number; 268). All experiments were performed according to the approved guidelines.

### 2.2. Wada Test

Under subcutaneous local anesthesia, a guide sheath was placed in the right femoral artery and the catheter was cannulated up to the level of the craniocervical portion of each internal carotid artery. To produce a contralateral hemiplegia and develop a theta–delta EEG pattern in the studied hemisphere, propofol was intraarterially injected, and this was approved by the institutional ethics committees. The dose was adjusted according to the body weight of each patient. Language testing began immediately after contralateral arm paresis and EEG changes. First, the counting and naming of dates and addresses and the naming of parents were used to evaluate expressive language functions. Subsequently, we examined visual naming, sentence reading, and sentence repetition. The maximum time required for the test was less than 5 min. Performance on these tests was scored as either normal or mildly, moderately, or severely deficient in estimating language lateralization, depending on the degree of impairment observed during right and left hemisphere injections. Estimated language lateralization was categorized into three patterns. The language-dominant hemisphere had the best score. The dominance of the language hemisphere could not be determined when there was no difference in scores.

If the test needed to be terminated prematurely, the side ipsilateral to the epilepsy focus was tested first. Simultaneous continuous EEG recordings excluded epileptic seizures that could interfere with the evaluation. This procedure was repeated on the contralateral side for a minimum of 15 min. No sedative drugs other than propofol were used throughout the testing.

### 2.3. fMRI Experiments

Each fMRI experiment was conducted on a different day from that of the Wada test. In each experiment, the patients were verbally instructed to close their eyes and carefully attend to the auditory stimuli presented using MRI-compatible noise-canceling headphones (Resonance Technology, Inc., Northridge, CA, USA). The stimulus intensity remained constant at 90 dB during the trials. Each trial comprised eight segments, alternating between four test segments and four control segments. No medical sedation was given during the scanning process.

**Figure 1:**
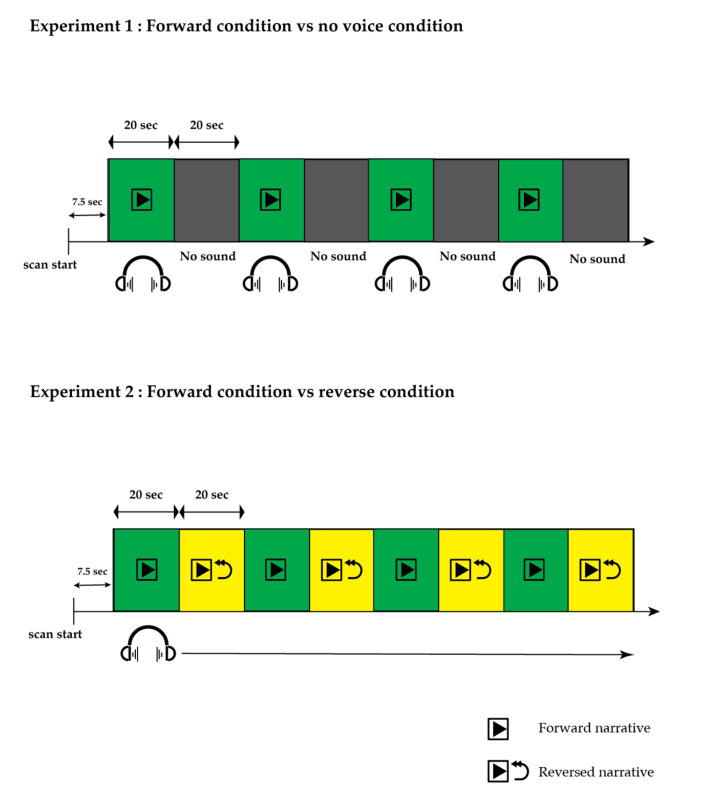
**Task timing in the functional magnetic resonance imaging experiments.** In Experiment 1, aimed at observing the overall activation elicited by listening to a narrative, an 80 s narrative was presented during four test segments (segments 1, 3, 5, and 7), with no auditory stimuli during the control segments. In Experiment 2, designed to eliminate activation induced by auditory voice processing in general, the narrative was delivered during the test segments, while the preceding test segments were played in reverse during each control segment.

Two experiments were carried out (Figure 1). In Experiment 1 (referred to as the FN task, involving forward narrative and no voice condition), we explored the overall activation pattern induced by listening to an 80 s narrative during each of the four test segments (segments 1, 3, 5, and 7). Throughout the control segments, no auditory stimuli were presented, and only attenuated machine noise was presented. The FN task was also used for the quality assurance check. We monitored the EPI map online; if the EPI map during the FN task showed no activation voxels, the experiments were stopped, and we checked the participants’ status and the measuring environment. If repeated failures were found even after checking all materials, we aborted that patient’s results. The absence of this sensory-specific activation is a good indication that the resulting activation map is inconsistent with the fMRI paradigm and that this case may not be suitable for correctly defining language-specific activation in a clinical setting [22]. Experiment 2 (referred to as the FR task, involving forward narrative and time-reversed narrative) was conducted to eliminate the activation resulting from auditory voice processing. In this trial, the narrative was presented during the test segments, while the preceding test segments were played in reverse during each control segment. Digitally recorded narratives of a news story spoken by a male Japanese speaker were employed in both experiments. The sound sequences for Experiments 1 and 2 were generated using PsychoPy (version 3.1) [23].

### 2.4. Image Data Acquisition and Analyses

The imaging procedures utilized a 3T Skyra MRI system (Siemens, Munich, Germany). Initially, sagittal anatomical images were obtained using a fast-spoiled gradient-recalled sequence with the following parameters: repetition time of 1800 ms, echo time of 2.03 ms, field of view of 230 × 230 mm, slice thickness of 0.90 mm, and a matrix of 128 × 128. This resulted in three-dimensional brain images. Subsequently, gradient echo planar imaging acquired 70 images per slice with a repetition time of 2500 ms, echo time of 30 ms, field of view of 192 × 192 mm, slice thickness of 4.0 mm, and a matrix of 64 × 64. We collected thirty slices for each brain sample. Data preprocessing and analysis were conducted using SPM12 in MATLAB (R2018b, MathWorks, Natick, MA, USA). In realignment preprocessing, the acquired data were reoriented relative to the anterior and posterior commissures. In spatial preprocessing, the participant’s head motions were corrected, and co-registration was performed between the structural T1 image and functional imaging datasets for each participant. A normalization preprocessing, including segmentation, was performed using default tissue probability maps. Following the normalization procedure, we visually verified the absence of any distortions in shape for each image. The images underwent smoothing with an 8 mm full width at half maximum Gaussian kernel, and the analyses were conducted utilizing a generalized linear model that incorporated a canonical hemodynamic response function [24]. The movement parameters derived from realignment were additionally incorporated as covariates of non-interest. High-pass filtering, set with a cutoff period of 128 s, was applied to eliminate slow signal drifts in the time series. Acquired clusters were considered statistically significant if the *p*-value for family-wise error was less than 0.05 or the uncorrected *p*-value was less than 0.001. For baseline correction, an implicit mask covering the temporoparietal lobules was utilized.

### 2.5. Lateralization Index (LI)

Language cortex distribution has been studied in auditory language processing areas (supramarginal, angular, and superior temporal gyri and middle temporal gyri, including Brodmann areas 21, 22, 37, 39, 40, 41, and 42) using LI to quantify the degree of lateralization of the blood oxygen level-dependent (BOLD) signal [25,26]. It was calculated using the following formula LI = (VL − VR)/(VL + VR), where VL and VR denote the number of active voxels for the left and right hemispheres. In this analysis, we set an extent threshold of >10 voxels. The LI ranged from −1 to +1, and language lateralization was categorized into three patterns. The LI less than −0.2 indicated right-sided lateralization, whereas LI greater than 0.2 was regarded as left-sided lateralization. An intermediate value (−0.2 to 0.2) was considered bilateral or mixed dominance.

## 3. Results

In this study, we utilized two levels of speech comprehension tasks for all 21 patients to assess language lateralization and identify specific brain cortices associated with language comprehension. Within this group, three patients exhibited low intellectual ability (full-test IQ < 50), and three had moderate intellectual disability (full-test IQ < 70). Subsequently, eight patients underwent epileptic focal resection surgery following language evaluation, with none of them experiencing postoperative language deficits.

### 3.1. fMRI Results

**Figure 2:**
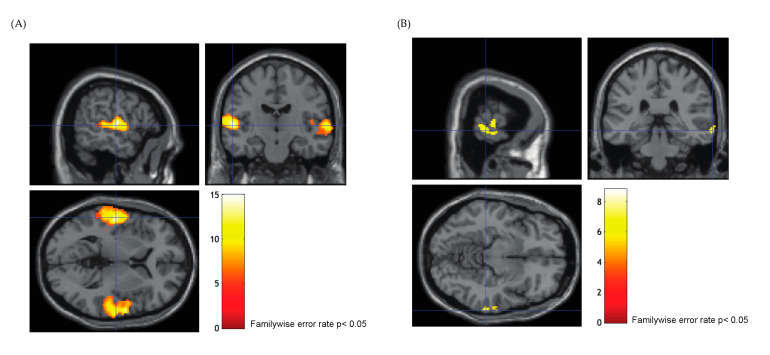
**Functional magnetic resonance imaging activation in the narrative forward condition.** The crosshair line on the images indicates the coordinate of the local maxima. (**A**) Significant activation distributed over the primary auditory cortex and superior temporal gyrus was found bilaterally in P1 (familywise error rate *p* < 0.05). (**B**) Unilateral activation was found in three patients (P5, P7, and P16), and auditory cortex activation appeared primarily in the right hemisphere in P16 (familywise error rate *p* < 0.05).

**Figure 3:**
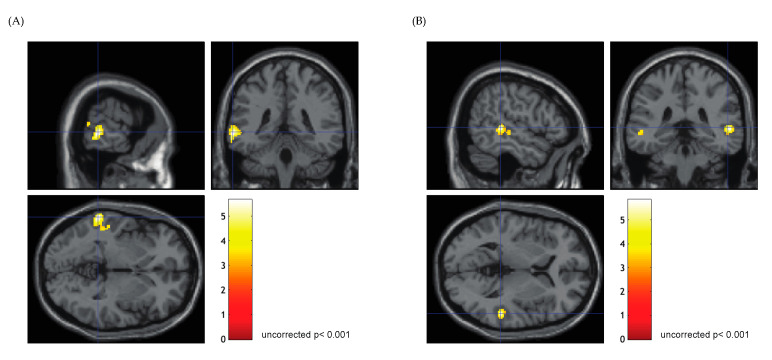
**Functional magnetic resonance imaging activation in the time-reversed narrative condition.** The crosshair line on the images indicates the coordinate of the local maxima. (**A**) In eighteen patients, passive listening to time-reversed narrative elicited significant activation in the left middle temporal gyrus or temporoparietal cortex. Significant activation distributed over the left posterior middle temporal gyrus was found in P4 (uncorrected *p* < 0.001). (**B**) Bilateral but right dominant activation was found in two patients (P8 is shown).

In the FN task, significant activation distributed throughout the primary auditory cortex and superior temporal gyrus (STG) was evident in all patients (family wise error, *p* < 0.05; Figure 2). The majority of patients showed bilateral activation (85.7%, *n* = 18), whereas unilateral activation was found in three patients (P5, P7, and P16). The activation clusters found bilaterally in the superior temporal lobe were similar to the findings of a previous report [20,27]. Passive listening in the FR task elicited significant activation in the middle temporal gyrus (MTG) or temporoparietal cortex (TPC) in all participants (uncorrected *p* < 0.001; Figure 3), which is in agreement with a previous study that investigated posterior language areas [28]. Eighteen patients showed left-dominant activation, while right-lateralized activation was found in one patient (P17), and two patients showed mixed dominance (P8, P11). The overall fMRI results are summarized in Table 2.

### 3.2. Concordance between fMRI and Wada Test for Language Lateralization

As we expected that the FN task would mostly elicit bilateral activation in the STG and primary auditory cortex, which also agrees with previous studies [29], we assessed the validity of language lateralization by comparing the results of the FR task and Wada test. We used LI to measure each patient’s language laterality in the FR task. The concordance rate of overall results was 95.2%; only one patient (P8) showed discrepant results between the fMRI and Wada tests. In the Wada test, 19 patients showed left-dominant language function, and 2 patients demonstrated right or bilateral language dominance. In the FR task, eighteen patients showed left-dominant activation, two showed bilateral activation, and one showed right dominant activation. In testing the concordance between fMRI and the Wada test protocol, the Kappa coefficient (Cohen’s Kappa) was 0.78 for language lateralization. In relation to the location of the epileptic focus, among the 10 patients with a left-sided epileptic focus, 1 was identified as right-dominant. However, no significant correlation was observed between epileptic foci and language lateralization. The comprehensive results are summarized in Table 3.

## 4. Discussion

In the present study, we applied fMRI tasks utilizing passive auditory stimuli to evaluate language lateralization in Japanese-speaking patients with drug-resistant temporal lobe epilepsy for presurgical evaluations. We used LI to determine language lateralization and successfully achieved favorable concordance with the results of the Wada test. To the best of our knowledge, this is the first article to evaluate language functions in Japanese-speaking patients with epilepsy using passive auditory language tasks.

We found that our fMRI method was easily applicable to participants with intellectual disabilities and demonstrated a reasonable level of concordance rate with the Wada test when compared to participants with relatively normal intelligence. Most language paradigms in fMRI for evaluating language functions are active paradigms, which require active participation and are designed for individuals with an average IQ. Therefore, participants with intellectual disabilities (defined as an IQ score below 70) or pediatric participants are considered a contraindication for language fMRI due to the challenges in following written instructions and completing tasks as intended [30]. They are typically unable to comply with complicated functional mapping protocols, which are invasive clinical gold standards, as well as non-invasive imaging techniques that are more easily used in normal adult populations. Passive fMRI paradigms can be conducted quickly and easily (requiring only a few minutes of scanning without the need for the patient’s active participation), making them ideal for use in participants who may not be expected to comply with more complex behavioral tasks. However, its weakness lies in its lower detection power compared to the active fMRI paradigms such as the naming task or verb generation task [31]. In terms of detection power, our fMRI paradigm has a notable strength as it has been validated to detect activations in language-related cortices even in patients with severe disorders of consciousness [20]. Suarez et al. demonstrated that a passive language fMRI task, using the auditory presentation of story listening, successfully evoked neural responses from the posterior language area in 15 pediatric patients, which agreed with Wada test results at 80% congruency [22]. They also reported that their passive task produced left-lateralized activation in the temporoparietal region compared with the active task, which is consistent with our results.

Many previous studies have compared the Wada test with language fMRI to evaluate the utility of fMRI in clinical settings for language lateralization. The latest meta-analysis for the concordance of fMRI and the Wada test reported an average of 85.4% (95% confidence interval 82.8–87.6%), but with a wide range of variability from 60.5% to a high of 100% [32]. The variations in results may be due to a difference in sensitivity between the Wada test and fMRI results. fMRI has high sensitivity and can detect all areas involved in language function. Janecek et al. reported that the more the LI in fMRI showed an atypical language distribution, the lower the agreement with the Wada test [33]. Consistent with their findings, one patient (P8) showed discrepant results between the fMRI and Wada tests in this study. In the fMRI assessment, he showed bilateral activation in the posterior language area in the FR task, which was determined to be mixed dominance (LI = −0.08), while he showed a left-lateralized response in the Wada test. P8 showed the lowest absolute LI value among all participants in the fMRI assessment, indicating that P8 has an atypical language distribution. Considering that the Wada test can be unreliable in determining the localization of language areas in cases with atypical language distribution [32,34], fMRI assessment is more likely to show the correct language distribution in this case.

In fMRI analysis, factors that may cause inconsistency or poor results include the choice of language task, ROI selection, statistical threshold setting, head motion noise, and falling asleep [35]. In passive auditory tasks, patient-side problems, such as the patient falling asleep or not hearing a speech sound, also significantly affected the results. Online quality check assurance is essential for practical use; however, it is difficult to confirm a patient’s level of consciousness during experiments. To address this issue, we designed tasks to assess the two levels of speech comprehension. In our FN task, we aimed to elicit auditory sensory-specific activation, and it was checked online during the experiment. If a participant did not show typical activation in the cortex, it would suggest that the participant was not in a condition to evaluate language function, such as falling asleep, or had an atypical brain structure unsuitable for standardized language assessment. Close consideration should be given to non-verbal activation patterns that are not directly related to language processing but are necessary for the successful and consistent application of a particular fMRI language protocol.

One of the limitations of the present study was the use of an implicit mask for fMRI analysis. Subtraction schemes were proposed to remove nonverbal activation, usually through carefully defined baseline conditions and subtraction methods in previous studies [36,37]. In this study, we used implicit masks, including temporoparietal lobules, for baseline correction in the FN and FR tasks. However, the ways to define the most appropriate choice of baseline conditions to eliminate all nonverbal activation without affecting the specific activation patterns of language function remain unclear. Further investigation should be conducted to confirm whether nonverbal activation is irrelevant when analyzing language-specific activation patterns. It is essential to acknowledge that our study might be prone to certain biases due to the relatively small sample size. The limited number of participants might not fully represent the broader population, which could affect the generalizability of our findings. To address this limitation in future studies, we aim to prospectively recruit a larger number of participants to enhance the robustness of our methods.

## 5. Conclusions

In this study, we reported a promising noninvasive fMRI-based method using passive auditory stimuli to evaluate language lateralization in Japanese-speaking patients with drug-resistant TLE, including those with intellectual impairment. This passive fMRI paradigm offers an alternative to invasive techniques like the Wada test and presents a reliable means of preoperative examination for language lateralization in patients undergoing epileptic surgery. This study represents an important step toward improving the safe and easy accessibility of language evaluation, particularly for individuals who have difficulty participating in active paradigms.

## Figures and Tables

**Table 1:** Patients’ characteristics.

	Sex	Age	Handedness	History of CNS Infection	Psychiatric Comorbidity	MMSE	VIQ	PIQ	FIQ	Side of Epileptic Focus	Seizure Semiology	Disease Onset [year]	Disease Duration [year]	Seizure Frequency [month]	Numbers of AEDs
P1	F	39	R	None	None	29	96	76	86	L	FIAS	28	11	3	2
P2	F	46	R	None	None	30	99	106	102	L	FIAS, FBTCS	28	18	2	3
P3	M	40	R	None	None	29	73	76	72	R	FIAS	24	16	4	3
P4	F	32	R	None	Yes	28	73	97	83	L	FIAS	29	3	6	3
P5	M	17	R	None	None	25	65	61	61	R	FIAS	7	10	18	4
P6	F	27	R	Yes	None	16	50	45	43	undetermined	FIAS, FAS	4	23	11	3
P7	F	34	R	None	None	30	76	76	74	L	FIAS, FBTCS	7	27	8	3
P8	M	47	L	None	None	30	111	116	114	L	FIAS, FBTCS	22	25	1	2
P9	F	35	R	None	Yes	27	65	75	67	R	FIAS	16	19	3	2
P10	F	14	L	None	None	30	92	80	92	L	FIAS	10	4	2	2
P11	F	45	R	Yes	Yes	20	61	51	53	R	FIAS	4	41	3	3
P12	F	44	R	Yes	None	29	84	79	80	L	FIAS	42	2	1	2
P13	M	45	R	None	Yes	23	55	46	47	undetermined	FIAS	6	39	6	4
P14	M	50	Bilateral	None	None	30	109	88	100	L	FIAS, FAS	45	5	8	3
P15	F	43	R	Yes	Yes	29	80	76	76	R	FIAS	32	11	3	2
P16	F	61	R	Yes	Yes	27	73	84	76	L	FIAS, FBTCS	3	58	5	2
P17	M	32	R	None	None	18	48	51	45	L	FIAS, FAS	13	19	44	5
P18	M	24	R	None	None	29	98	88	96	R	FIAS, FAS	13	11	3	3
P19	F	61	R	None	Yes	26	113	76	89	R	FIAS, FBTCS	29	32	10	2
P20	F	36	R	None	None	25	74	87	84	R	FIAS, FAS	32	4	1	2
P21	M	38	R	None	None	28	77	80	72	R	FIAS	22	16	4	3
mean		38.6				26.6	79.6	76.9	76.8			19.8	18.8	7.0	

F: Female, M: Male, L: Left, R: Right, FAS: Focal awareness seizure, FIAS: Focal impaired awareness seizure, FBTCS: Focal bilateral tonic clonic seizure, CNS: Central nervous system, MMSE: Mini-Mental State Examination, VIQ: Verbal IQ, PIQ: Performance IQ, FIQ: Full Scale IQ, AEDs: Anti-epileptic drugs.

**Table 2:** Functional magnetic resonance imaging results in FN and FR task.

	FN Task			FR Task				
	Numbers of Activated Voxels		LI	Numbers of Activated Voxels		LI	T Max	T Max Coordinate
	Left	Right		Left	Right			
P1	1339	1295	0.02	272	90	0.50	4.39	−54, −48, 28
P2	1954	1046	0.30	1096	0	1.00	9.67	−64, −28, −2
P3	1532	1706	−0.05	278	47	0.71	4.29	−56, −40, 2
P4	931	751	0.11	262	0	1.00	5.65	−64, −40, −2
P5	43	0	1.00	25	0	1.00	3.8	−48, −36, −2
P6	966	222	0.63	869	84	0.82	4.38	−52, −12, −16
P7	246	0	1.00	238	67	0.56	4.63	−66, −46, 4
P8	1617	2897	−0.28	102	120	−0.08	4.93	54, −36, 4
P9	2101	2905	−0.16	627	0	1.00	5.7	−62, −46, 8
P10	312	342	−0.05	86	0	1.00	3.37	−32, −60, 58
P11	574	1263	−0.38	564	561	0.00	6.14	−32, −68, 30
P12	2243	2247	0.00	121	0	1.00	4.51	−54, −34, −6
P13	626	1079	−0.27	216	16	0.86	4.2	−54, −54, 0
P14	168	144	0.08	103	20	0.67	4.47	−52, −52, 20
P15	1226	2161	−0.28	1378	0	1.00	6.11	−36, −50, 44
P16	0	193	−1.00	35	0	1.00	4.3	−62, −44, 0
P17	618	598	0.02	0	16	−1.00	3.16	66, −42, 10
P18	673	379	0.28	313	0	1.00	4.01	−62, −52, 20
P19	502	171	0.49	417	31	0.86	6.48	−44, −40, 46
P20	399	699	−0.27	334	11	0.94	5.15	−40, −52, 50
P21	2415	1351	0.28	58	0	1.00	3.75	−60, −42, −2

FN, forward narrative and no voice condition; FR, forward narrative and time-reversed narrative.

**Table 3:** Results of Wada test and FR task in functional magnetic resonance imaging.

	Estimated Epileptic Focus	Result of Wada Test	Result of FR Task
P1	L	L	L
P2	L	L	L
P3	R	L	L
P4	L	L	L
P5	R	L	L
P6	undetermined	L	L
P7	L	L	L
P8	L	L	Bilateral
P9	R	L	L
P10	L	L	L
P11	R	Bilateral	Bilateral
P12	L	L	L
P13	undetermined	L	L
P14	L	L	L
P15	R	L	L
P16	L	L	L
P17	L	R	R
P18	R	L	L
P19	R	L	L
P20	R	L	L
P21	R	L	L

L: Left; R: Right; FR: forward narrative and time-reversed narrative.

## Data Availability

The data supporting the study findings are available upon reasonable request from the corresponding author in accordance with the data policies. The data are not publicly available because they contain information that can compromise the consent of the research participants.
